# Individual Differences in Self-Talk Frequency: Social Isolation and Cognitive Disruption

**DOI:** 10.3389/fpsyg.2019.01088

**Published:** 2019-05-10

**Authors:** Thomas M. Brinthaupt

**Affiliations:** Middle Tennessee State University, Murfreesboro, TN, United States

**Keywords:** self-talk, intrapersonal communication, self-talk scale, social isolation, cognitive disruption

## Abstract

Despite the popularity of research on intrapersonal communication across many disciplines, there has been little attention devoted to the factors that might account for individual differences in talking to oneself. In this paper, I explore two possible explanations for why people might differ in the frequency of their self-talk. According to the “social isolation” hypothesis, spending more time alone or having socially isolating experiences will be associated with increased self-talk. According to the “cognitive disruption” hypothesis, having self-related experiences that are cognitively disruptive will be associated with increased self-talk frequency. Several studies using the Self-Talk Scale are pertinent to these hypotheses. The results indicate good support for the social isolation hypothesis and strong support for the cognitive disruption hypothesis. I conclude the paper with a wide range of implications for future research on individual differences in self-talk and other kinds of intrapersonal communication.

Several researchers have studied individual differences in the frequency of intrapersonal communication (e.g., Honeycutt, 2010; Morin et al., 2011; Hurlburt et al., 2013; Ren et al., 2016). It is clear that people differ in how often they typically talk to themselves. What is less clear are the factors that might account for such individual differences in intrapersonal communication. Considering these factors is likely to have implications for a wide range of research and practice domains. For example, cognitive-behavioral interventions (e.g., Hollon and Beck, 2013) may be more (or less) effective for frequent compared to infrequent self-talkers. Sport psychologists who are interested in enhancing athletic performance through self-talk manipulations (e.g., Hatzigeorgiadis, 2006) might improve their efforts by taking into account individual differences in self-talk frequency. Educational practices that utilize self-talk as a self-regulatory tool (e.g., Deniz, 2009) could be adjusted based on how frequently or infrequently students talk to themselves.

In this paper, I examine two potential sources of individual differences in self-talk frequency. These sources focus on the potential interpersonal aspects of self-talk (i.e., how different kinds of social experiences might relate to its frequency) and how a variety of intrapersonal events (such as cognitive, perceptual, and sensory experiences) might relate to self-talk frequency. First, I define self-talk as a category of intrapersonal communication and examine the various self-regulatory functions that it serves. Next, I review the characteristics and research examining the psychometric properties of the Self-Talk Scale (STS; Brinthaupt et al., 2009), a measure designed to assess self-talk frequency. In the next sections of the paper, I examine the findings that are pertinent to the “social isolation” and “cognitive disruption” hypotheses of individual differences in self-talk. I conclude the paper with recommendations for how to test these hypotheses further using the STS and related measures. Implications for future research on self-talk and intrapersonal communication frequency are also presented.

## Self-Talk and Other Kinds of Intrapersonal Communication

As the current Research Topic contributors and others illustrate, the research literature on intrapersonal communication is alive and well. Among the varieties of this kind of communication are silent self-talk (inner speech; McCarthy-Jones and Fernyhough, 2011), out loud self-talk (private speech; Duncan and Cheyne, 1999), internal dialogues (Hermans, 1996), auditory imagery (MacKay, 1992), and self-statements (Kendall et al., 1989). Researchers and reviewers have identified a wide range of possible functions served by self-talk (Langland-Hassan and Vicente, 2018). For example, psychologists propose that self-talk plays a role in inhibiting impulses, guiding courses of actions, and monitoring goal progress (Mischel et al., 1996). Self-talk has also been conceived of as a “meta-monitoring” of behavior and goal progression that can affect emotional reactions and responses to behavioral deficits (Carver and Scheier, 1998).

Sport psychologists highlight the importance of instructional (e.g., giving directions) and motivational (e.g., psyching oneself up) self-talk as well as other kinds of intrapersonal communication with respect to sport or athletic performance (Hatzigeorgiadis et al., 2011; Latinjak et al., 2019). Clinical psychologists have long been interested in the content of self-talk, particularly whether it is positive or negative (Kendall et al., 1989) and whether what one says to oneself is maladaptive or dysfunctional (e.g., Ellis, 1962; Beck, 1976). Others (e.g., Fernyhough, 2016; Van Raalte et al., 2016) further differentiate between condensed/automatic and expanded/elaborated self-talk.

Fernyhough’s (2016) summary nicely captures many of the everyday self-regulatory functions served by self-talk: “[Self-talk] can help us to plan what we are about to do and to regulate a course of action once it has started; it can give us a boost in keeping information in mind about what we are supposed to be doing, and in psyching ourselves up for action in the first place. For many of us, it provides a central thread to our conscious experience and is integral to our sense that we have a coherent, enduring self” (p. 107).

In summary, conceptual and research distinctions focus on the audible/overt, automatic, affective, and conversational aspects of self-talk. Following these distinctions, I define self-talk as self-directed or self-referent speech (either silent or aloud) that serves a variety of self-regulatory and other functions. This broad definition is designed to capture some of the primary features of the general phenomenon of talking to oneself that are amenable to the study of individual differences.

## The Self-Talk Scale

The Self-Talk Scale (STS) (Brinthaupt et al., 2009) is a measure of the frequency with which individuals talk to themselves under a variety of circumstances. It assumes a functional approach by measuring how often people talk to themselves (silently or aloud) in response to specific events or situations. The STS measures four specific self-talk functions: self-criticism, self-reinforcement, self-management, and social-assessment. Respondents rate the 16 STS items with a 5-point frequency scale (1 = *never*, 5 = *very often*) and using the common stem “I talk to myself when…” Self-critical self-talk assesses negative events (e.g., when something bad has happened or when feeling ashamed of something one has done). Self-reinforcing self-talk refers to positive events (e.g., when feeling happy for oneself or proud of something one has done). Self-managing self-talk measures general self-regulation (e.g., when mentally exploring a possible course of action or when giving oneself directions or instructions about what to do or say). Finally, social-assessing self-talk applies to people’s social interactions (e.g., when replaying something one has said to another person or analyzing something that someone recently said).

In our research, we find that total STS scores are normally distributed among college student samples. Test-retest stability of total scores (over 3 months) is good (i.e., *r* = 0.69; Brinthaupt et al., 2009, Study 7). Total and subscale internal consistencies are good (i.e., in the 0.85–0.94 range). We typically conduct correlational research using total and subscale STS scores (e.g., Brinthaupt et al., 2009, Study 4; Shi et al., 2015) as well as compare infrequent (lower 25%) with frequent (upper 25%) STS groups on a variety of measures (e.g., Brinthaupt et al., 2015, Study 2; Brinthaupt et al., 2009, Study 5).

Rasch analysis has supported the use of the STS response format and the use of the STS total score as a unidimensional measure of self-talk frequency (Brinthaupt and Kang, 2014). Brinthaupt et al. (2015) found that the self-talk situations included in the STS are frequently reported occurrences in people’s lives and that STS scores (from 6 weeks earlier) were significantly related to reports of self-talk in response to relevant situations that had occurred over the past 2 days (*r* = 0.45; Study 1). We also found, in a week-long experience sampling study, that frequent self-talkers (measured one month earlier) reported talking to themselves significantly more often during recent events over the past 2 h compared to infrequent self-talkers (*d* = 0.83; Study 2). Qualitative (open-ended) research on when, where, and why people talk to themselves supports the four STS subscales/functions (e.g., Morin et al., 2018). Finally, there is good cross-cultural support for the structure and properties of the STS (e.g., Khodayarifard et al., 2014; Ren et al., 2016).

In summary, as a measure of individual differences in self-talk frequency, the structure and properties of the STS have been well supported. Although research indicates wide individual variation in the frequency of self-talk, there are few systematic assessments of the possible factors that might account for why people differ in their self-talk frequency. In the following sections, I present two hypotheses that are informed by our and others’ research using the STS.

## The Social Isolation Hypothesis

One potential reason for why people differ in their self-talk frequency is the extent of their social isolation. Research shows that social isolation is a significant risk factor for physical and mental health (Cacioppo and Cacioppo, 2014). In addition, the frequency of self-referential pronoun use is positively associated with a variety of socially isolating physical and mental illnesses (Fineberg et al., 2016). According to this hypothesis, individuals who spend more time alone or who have more socially isolating experiences will report more frequent self-talk. The rationale here is that people may be motivated to create or manage their “social” interactions (*via* self-talk) when their social experiences are limited or unsatisfactory. Several published studies are pertinent to this hypothesis.

Some research has examined how childhood social experiences might be associated with differences in self-talk frequency. For example, adult only-children report significantly higher levels of overall (*d* = 0.28) and self-critical (*d* = 0.46) self-talk frequency than sibling children (Brinthaupt and Dove, 2012, Study 1). Adults who report having had an imaginary companion in childhood report significantly higher levels of overall (*d* = 0.16), self-reinforcing (*d* = 0.23), and self-managing (*d* = 0.17) self-talk frequency than those who did not have an imaginary companion (Brinthaupt and Dove, 2012, Study 2). We speculated that only children may be more comfortable being alone, more likely to engage in self-socialization, and more self-focused and autonomous compared to children with siblings. Having an imaginary companion in childhood might be associated with greater use of imagery, increased awareness of internal states, and being more creative and fantasy-prone compared to not having had such an experience. These factors might play a role in determining the levels of self-talk frequency in both childhood and adulthood.

In a study of loneliness, self-talk, and well-being using a German adult sample, Reichl et al. (2013) found that need to belong (*r* = 0.26) and loneliness (*r* = 0.29) scores were positively correlated with overall self-talk frequency, with similar relationships for all of the STS subscales. They also found higher negative correlations between loneliness and mental health for frequent compared to infrequent self-talkers. These results pertain directly to the rationale for the social isolation hypothesis, indicating that having limited or unsatisfactory social relationships was associated with increased self-talk frequency.

Other research has studied social-related variables and their relationship to STS scores. For example, using a Persian translation of the STS, Akbari-Zardkhaneh et al. (2018) found that extraversion scores were negatively related to the frequency of self-managing self-talk (*r* = −0.29) and that insensitivity scores (e.g., being unwilling to accept other people’s opinions) were negatively related to self-critical self-talk frequency (*r* = −0.27). In other words, people who are more introverted tended to report more self-managing self-talk, whereas people who do not believe that they are superior to other people (lower insensitivity; Van Kampen, 2000) reported higher levels of self-critical self-talk.

In summary, there is good support for the social isolation hypothesis, with a consistent pattern across the studies, and most effect sizes in the small range. It is clear that certain features of social experiences (e.g., having limited or unsatisfactory relationships) are associated with increased levels of self-talk frequency. Further systematic assessment of the social isolation hypothesis is needed. For example, researchers could examine fear of negative evaluation (e.g., Tanaka and Ikegami, 2015), shyness (e.g., Tang et al., 2017), and social anxiety disorder (e.g., Poole et al., 2017). According to the social isolation hypothesis, each of these characteristics should relate positively to overall self-talk frequency as well as to the self-critical facet of self-talk frequency, based on the findings of previous research.

Exploring different kinds of internal dialogues (e.g., Oleś, 2009) might also help to assess the validity of the social isolation hypothesis. For example, integrative dialogues (i.e., internal conversations that resolve opposing views or reduce self-discrepancies) might be characterized by high levels of self-reinforcing, self-managing, and social-assessing self-talk, whereas confrontational dialogues (i.e., those that create internal dissonance or favor one viewpoint over another) might be characterized by high levels of self-critical and self-managing self-talk (Puchalska-Wasyl, 2017). The “helpless child” interlocutor identified by Puchalska-Wasyl (2015) should be associated with frequent self-critical self-talk, as it is characterized by feelings of powerlessness and isolation.

Finally, it would be interesting to explore self-talk frequency with respect to other facets of social isolation, such as being socially disconnected, living alone or with pets, and having recently suffered the termination of a romantic relationship. Individuals experiencing such short- or long-term social features might be motivated to compensate for their limited or unsatisfactory experiences through increased levels of overall or specific kinds of self-talk. For example, researchers could measure self-talk levels of participants before and after they experience a socially isolating event. Investigators might also expose participants to hypothetical threats to or affirmations of their social connections and assess the content and frequency of self-talk in response to those manipulations.

## The Cognitive Disruption Hypothesis

Cognitive disruption related to the need to explain or understand personal events or experiences is another potential reason for individual differences in self-talk frequency. Research shows that people who experience cognitive disruption following negative or stressful events demonstrate performance and self-regulatory decrements (e.g., Gunther et al., 2007; Helton et al., 2011). According to this hypothesis, self-related experiences that are cognitively disruptive (such as anxiety, obsessive-compulsive tendencies, and schizotypy) will be associated with increased self-talk frequency. The rationale here is that having anomalous, upsetting, or disturbing self-related experiences should press a person into trying to resolve, understand, or clarify those experiences. Self-talk is one self-regulatory tool that is predicted to be used under these circumstances. There are several research studies using the STS that are pertinent to this hypothesis.

Large percentages of people report that they feeling anxious about speaking in public (e.g., Stein et al. 1996). Because of its prominence, anxiety about public speaking is an excellent case for studying the relationship between self-talk and the cognitive disruptions caused by anxiety. Research conducted by Shi et al. (2015) examined whether individuals who were anxious about delivering a forthcoming public speech reported more self-talk related to that speech. Just prior to delivering their speech, college student participants completed the STS (adapted to their speech preparation) and a measure of public speaking anxiety (PSA). The results showed that self-critical (*β* = 0.15) and social-assessing (*β* = 0.31) self-talk were positively related to PSA, whereas self-reinforcing self-talk was negatively related to PSA (*β* = −0.28). We interpreted these results to suggest that individuals with high PSA were cognitively “busier” than those with low anxiety as they prepared for their upcoming speech. In a follow-up study (Shi et al., 2017), we found that self-managing self-talk was positively associated with the rated organization of an actual speech (*r* = 0.23) and that PSA mediated the effects of self-critical and social-assessing self-talk on rated speech delivery, with self-critical self-talk indirectly decreasing speech delivery scores through its influence on increasing speakers’ PSA levels.

Research shows that people normally have a variety of intrusive and ruminative thoughts and that these thoughts can sometimes develop into the serious clinical obsessions that characterize obsessive-compulsive disorder (e.g., Mancini et al., 1999). Studies also show that obsessional, compulsive tendencies are associated with an over-awareness of self-processes (e.g., Baumeister and Heatherton, 1996). Thus, it seems reasonable that obsessive-compulsive tendencies might be related to increased self-talk frequency. Research using the STS supports this line of reasoning. For example, compared to infrequent self-talkers, frequent self-talkers report higher levels of obsessive-compulsive tendencies (*d* = 0.80), in particular, impaired control over mental activities (*d* = 0.77) and checking behaviors (*d* = 0.83) (Brinthaupt et al., 2009; Study 5). Khodayarifard et al. (2014) also found moderate positive correlations (i.e., in the 0.32–0.34 range) between obsessive-compulsive tendencies and overall and subscale self-talk frequency.

Another kind of self-related cognitive disruption is associated with the occurrence of schizotypy tendencies, which are milder forms and predictors of schizophrenia (e.g., Kwapil et al., 2018). Schizophrenia and schizotypy have long been considered to be disorders of the self by researchers and theorists, and a variety of self-related impairments and self-experience anomalies have been reported by those with schizotypy tendencies (Parnas, 2003). In a recent study using the STS (Brinthaupt, Smartt, and Long, under review), we found that positive (e.g., thought disruptions, perceptual anomalies) and disorganized (e.g., disruptions of current behavior, situational confusion) schizotypy factors were positively and significantly correlated with self-talk factors (*r*s in the 0.28–0.44 range), but that negative schizotypy factors (e.g., speech impairments, diminished reactivity and affect) were unrelated to self-talk frequency. We interpreted these results as consistent with a “self-regulatory focus” explanation rather than reflecting self-regulatory or intrapersonal deficits.

There are additional studies that are pertinent to the cognitive disruption hypothesis. Using a Chinese college student sample, Ren et al. (2016) found significant relationships between impulsivity and self-talk frequency. In particular, motor impulsiveness scores (e.g., doing things without thinking) were positively related to self-critical self-talk (*r* = 0.31), whereas cognitive impulsiveness scores (e.g., making quick cognitive decisions) were negatively related to self-reinforcing self-talk (*r* = −0.27). Indirect support for the cognitive disruption hypothesis comes from research that examines general cognitive variables and their relationship to self-talk frequency. For example, overall self-talk frequency is positively correlated with scores on private self-consciousness (*r* = 0.37) and using verbal information processing strategies (*r* = 0.47), and people who report frequent self-talk show higher need for cognition scores than do infrequent self-talkers (*d* = 0.64) (Brinthaupt et al., 2009, Studies 4–6). Furthermore, Ren et al. (2016) found that self-managing self-talk was positively but weakly correlated with a variety of reasoning and working memory tasks (*r*s in the 0.16–0.22 range).

In summary, there is strong support for the cognitive disruption hypothesis, with moderate-to-large effect sizes reported in the research literature. A variety of self-related and general cognitive measures are associated with increased levels of overall or subscale self-talk frequency. If the cognitive disruption hypothesis is accurate, it is likely that other kinds of self-related disruption, such as identity disturbance (e.g., Kaufman et al., 2015), will be associated with increases in self-talk frequency. Conducting experimental manipulations would be the best way to provide direct support for the cognitive disruption hypothesis. For example, researchers might create situations that result in anomalous perceptual or sensory experiences and then monitor overt and covert self-talk as participants attempt to explain or understand those experiences.

Other examples of relevant cognitive disruption might include dissociative experiences (e.g., Alderson-Day et al., 2018), perfectionism (e.g., Moore et al., 2018), and academic procrastination (e.g., Grunschel et al., 2016). In each of these cases, higher overall or subscale scores (particularly the self-critical facet of self-talk) would be expected to be associated with increased self-talk frequency. For example, research shows that perfectionism is associated with increased levels of stress and stress reactivity (Flett et al., 2016) as well as increased intrusive imagery and difficulty completing tasks (Lee et al., 2011). Such tendencies should increase the need for self-regulatory self-talk. To date, no research has examined these possibilities.

## Other Possible Factors Related to Individual Differences in Self-Talk Frequency

This paper reports research that examines the relationship of personality and personal experience factors to self-talk frequency. There are likely to be shorter-term, less stable factors that might affect when, where, and how much one talks to oneself (Hardy et al., 2009). For example, it is possible that unstable, situational experiences of social isolation or disruption (e.g., experiencing anger or rejection from a friend or family member) or cognitive disruption (e.g., experiencing an acute stressful life event) will be associated with more frequent self-talk frequency, regardless of one’s normal levels of self-talk. Future research could explore these possibilities. Sport psychology appears to be particularly well-equipped to test many of these ideas.

Although the results reported here do not directly assess this possibility, it appears likely that self-regulatory disruptions (e.g., Baumeister and Heatherton, 1996) will precipitate increased self-talk. For example, disruption of plans, failure to engage in desirable behaviors or to stop engaging in undesirable behaviors, and having difficulty meeting one’s internalized standards should all increase the need to engage in the self-regulatory functions served by self-talk. Future research could explore these possibilities as well. Conducting research along the lines described here will help to clarify the extent that self-talk frequency differs based on stable, individual differences and as a response to short-term events and experiences.

Future research should also contrast the social isolation and cognitive disruption hypotheses. The results reported in this paper suggest that cognitive disruption is more strongly related to self-talk frequency than are socially isolating experiences. Brinthaupt et al. (under review) found that the interpersonal superordinate schizotypy facet was much less strongly related to self-talk frequency than were the cognitive-perceptual anomalies and disorganized thinking superordinate facets. This result provides an initial comparison of the relative strength of the social isolation and cognitive disruption hypotheses, with stronger support for the latter.

The social isolation and cognitive disruption hypotheses can be further tested using measures that include other varieties of inner speech (Alderson-Day et al., 2018) or dialogic functions (Puchalska-Wasyl, 2017) not assessed by the STS. As reported earlier, there is some evidence of a weak, positive relationship between extraversion and self-talk frequency. However, overall, the Big 5 personality traits appear to be weakly related to self-talk frequency. As Uttl et al. (2011) found, most measures of inner speech or self-talk show very weak relationships with the NEO traits. Thus, the issue is not one that is specific to the STS. Upon reflection, the need or desire to talk to oneself should not be specific to high or low levels of core personality traits. Being generally sociable, talkative, trusting, curious, organized, or distress-prone should not, per se, incline people to talk more or less frequently to themselves. People who are low versus high in agreeableness or openness will probably differ less in the frequency of their self-talk than in its content (e.g., its valence, whether it is more approach or avoidance in nature).

An additional hypothesis for individual differences in self-talk frequency might be that having emotionally disruptive experiences will precipitate the need for more self-talk. To date, there is some support for this “emotional disruption” hypothesis. For example, depression (e.g., Khodayarifard et al., 2014), self-esteem (e.g., Brinthaupt et al., 2009, Studies 4 and 6), and neuroticism (e.g., Uttl et al., 2011; Akbari-Zardkhaneh et al., 2018) are weakly related to overall self-talk frequency, but more strongly related to self-critical self-talk. Self-criticism has been identified as a trans-diagnostic process related to a variety of negative clinical outcomes (Shahar et al., 2012). Observational research of tennis players shows that negative self-talk increases in frequency following lost points during a competitive match (Van Raalte et al., 1994). Future research could explore whether experiencing negative emotions is most strongly associated with self-critical self-talk frequency, whereas experiencing positive emotions is most strongly associated with self-reinforcing (and possibly self-managing) self-talk frequency.

In conclusion, research exploring individual differences in self-talk frequency has uncovered moderate support for the social isolation hypothesis and strong support for the cognitive disruption hypothesis. As alluded to in the introduction, measuring individual differences in self-talk frequency has the potential to be useful and informative for a variety of therapeutic, sport, and educational interventions. It is conceivable that cognitive or behavioral interventions might “take” more easily and readily with individuals who frequently rather than infrequently talk to themselves. By using the Self-Talk Scale and related measures, researchers can examine these possibilities and a wide range of other interesting questions.

## Author Contributions

The author confirms being the sole contributor of this work and has approved it for publication.

### Conflict of Interest Statement

The author declares that the research was conducted in the absence of any commercial or financial relationships that could be construed as a potential conflict of interest.
